# 3,4-Dihydroxybenzalactone Suppresses Human Non-Small Cell Lung Carcinoma Cells Metastasis via Suppression of Epithelial to Mesenchymal Transition, ROS-Mediated PI3K/AKT/MAPK/MMP and NFκB Signaling Pathways

**DOI:** 10.3390/molecules22040537

**Published:** 2017-03-28

**Authors:** Wei Chao, Jeng-Shyan Deng, Pei-Ying Li, Yu-Chia Liang, Guan-Jhong Huang

**Affiliations:** 1 School of Chinese Pharmaceutical Sciences and Chinese Medicine Resources, College of Chinese Medicine, China Medical University, Taichung 404, Taiwan; sebrina0427@hotmail.com (W.C.); allen1987323@yahoo.com.tw (Y.-C.L.); 2 Department of Health and Nutrition Biotechnology, Asia University, Taichung 404, Taiwan; dengjs@asia.edu.tw; 3 School of Pharmacy, College of Pharmacy, China Medical University, Taichung 404, Taiwan; chacolate042276@yahoo.com.tw

**Keywords:** *Phellinus linteus*, DBL, cancer metastasis, MMPs, EMT, ROS

## Abstract

3,4-Dihydroxybenzalactone (DBL) was isolated from *Phellinus linteus* (PL), which is a folk medicine possessing various physiological effects. In this study, we used highly metastatic A549 cells to investigate efficacy of DBL inhibition of cancer metastasis and possible mechanisms. The results revealed DBL inhibited migratory and invasive abilities of cancer cells at noncytotoxic concentrations. We found DBL suppressed enzymatic activities, protein expression, and RNA levels of matrix metalloproteinase (MMP)-2 and MMP-9. Western blot results showed DBL decreased phosphoinositide 3-kinase (PI3K)/AKT, phosphorylation status of mitogen-activated protein kinases (MAPKs), and focal adhesion kinase (FAK)/paxillin, which correlated with cell migratory ability. DBL also affected epithelial to mesenchymal transition (EMT)-related biomarkers. In addition, DBL enhanced cytoprotective effects through elevated antioxidant enzymes including heme oxygenase 1 (HO-1), catalase, glutathione peroxidase (GPx), and superoxide dismutase (SOD). Moreover, DBL influenced the nuclear translocation of nuclear factor κB (NFκB), nuclear factor erythroid 2-related factor 2 (Nrf2), Snail, and Slug in A549 cells. Taken together, these results suggested that treatment with DBL may act as a potential candidate to inhibit lung cancer metastasis by inhibiting MMP-2 and -9 via affecting PI3K/AKT, MAPKs, FAK/paxillin, EMT/Snail and Slug, Nrf2/antioxidant enzymes, and NFκB signaling pathways.

## 1. Introduction

Lung cancer, especially non-small cell lung cancer (NSCLC), is one of the most lethal cancers in the world [1]. Despite there being multiple kinds of therapies—such as chemotherapy, radiotherapy, and so on—the mortality for lung cancer patients is still high [2]. Cancer metastasis is the main reason for treatment failure and death in cancer patients [3].

Cancer metastasis is a series complicated process, including cancer cell adhesion, migration, and angiogenesis, which are characteristics of metastasis [4,5]. Primary tumor cells need more space and nutrition; the metastatic cells change the cell phenotype (epithelial to mesenchymal transition, EMT), lose adhesion, invade the basement membrane or extracellular matrix (ECM), increase motility, and migrate through circulation systems to distant sites to form other tumors [6]. Therefore, prevention of cancer metastasis is a critical therapeutic strategy for lung cancer patients.

Reactive oxygen species (ROS)—including superoxide (O^2−^), hydroxyl radical (·OH), and H_2_O_2_—are constantly generated in aerobic organisms [7]. ROS act as inducers of matrix metalloproteinase (MMP) production and regulators in the EMT process, which are all related with cancer metastatic progression [7,8]. Excess intracellular ROS in microenvironments may trigger the activation of nuclear factor erythroid 2-related factor 2 (Nrf2) and downstream proteins such as heme oxygenase 1 (HO-1). For protecting the organism against harmful ROS, there are some enzymatic antioxidants (such as superoxide dismutase (SOD), glutathione peroxidase (GPx), and catalase) [9]. Therefore, there are more studies to investigate the relation between antioxidant and anticancer metastasis.

Besides, the essential role of cancer metastasis is proteolytic enzymes degrading the ECM. Among these enzymes, the MMPs—which are a family of zinc-dependent endopeptidase—participate in tumor cell invasion and metastasis [10,11]. The MMP family can be classified into five subgroups according to substrate specificity and structure, including collagenases, stromelysins, gelatinases, membrane-type MMPs, and other MMPs [12]. Of the MMPs, a specific subset are the gelatinases (MMP-2 and MMP-9), which have been the subject of research for their high levels of expression in various malignant tumors and close relation with cancer cell metastasis [13,14]. The regulation of MMP-2 and -9 expression is via various transcription factor such as nuclear factor κB (NFκB), mitogen-activated protein kinases (MAPKs), and phosphoinositide-3 kinase/protein kinase B (PI3K/AKT) pathways [15].

3,4-Dihydroxybenzalactone (DBL) is a component found only in fungi, especially in the *Phellinus* genus [16]. *Phellinus linteus* (PL), commonly called “sanghuang”, has been used as food and medicine in oriental countries. It contains many bioactive compounds and is known to improve health and prevent various diseases, including cancer [17]. DBL is a polyphenol compound and previous studies have indicated that it possesses many activities, such as antioxidant [18], anti-inflammatory [19], anti-Parkinson’s disease [20], and antitumor [16]. To date, there are no direct evidences indicating an inhibitory effect of DBL on lung cancer metastasis. In this study, we investigated the effect of anti-metastasis in vitro. Additionally, Western blot analysis was conducted to identify the related signaling pathways affected by DBL.

## 2. Results

### 2.1. The Chemical Profile and Cytotoxicity of DBL

#### 2.1.1. Isolation of DBL from *Phellinus linteus* and Its Structural Characterization

The fruiting body of *Phellinus linteus* (PL) was dried, partitioned, and obtained from the ethyl acetate fraction. Chromatographic patterns from HPLC analysis of this soluble fraction showed peaks corresponding to the retention times. The chemical structure was elucidated by NMR spectroscopy and mass spectrometry studies and was identified as DBL. The spectral data of the isolated substance was: yellow needles C_10_H_10_O_3_; ^1^H-NMR (DMSO, 400 MHz) δ 2.25 (s, 3H, CH_3_), 6.47 (d, 1H, *J* = 16 Hz, CH), 6.77 (d, 1H, *J* = 8.2 Hz, ArH), 6.98 (dd, 1H, *J* = 8.2, 2.0 Hz ArH), 7.05 (d, 1H, *J* = 2.0 Hz, ArH), 7.42 (d, 1H, *J* = 16 Hz, CH), 9.24 (s, 1H, OH), 9.62 (s, 1H, OH); ^13^C-NMR (100 MHz, DMSO) δ 27.5, 115.1, 116.2, 122.1, 124.3, 126.2, 144.5, 146.0, 148.8, 198.5.

#### 2.1.2. The Cell Viability of DBL in A549 Cells

**Figure 1:**
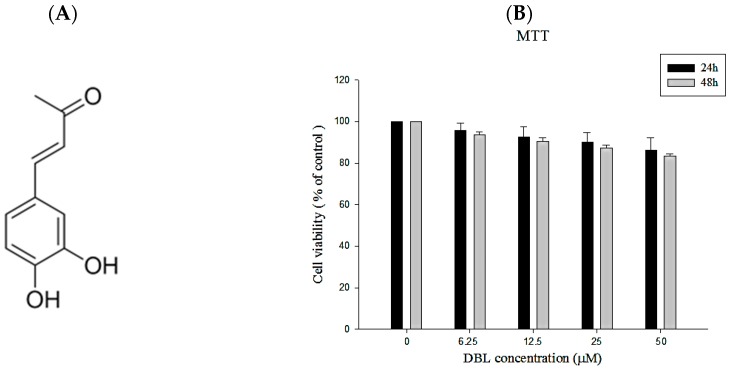
The chemical profile of dihydroxybenzalactone (DBL). (**A**) Chemical structure of DBL; (**B**) effects of DBL on cell viability in A549 cells for 24 and 48 h by MTT assay. A549 cells were treated with indicated concentrations (0, 6.25, 12.5, 25, 50 μM). Values represent mean ± SEM from three independent experiments.

First, we investigated the cytotoxicity of DBL in A549 cells though MTT assay. As shown in Figure 1B, there are no obvious cytotoxic effects in our present results. About 80% of cells were still alive after we treated them with 50 μM DBL for 48 h. Therefore, 0–50 μM of DBL was used for subsequent experiments.

### 2.2. DBL Inhibits Migration, Invasion, and Adhesion Ability of A549 Cells

**Figure 2:**
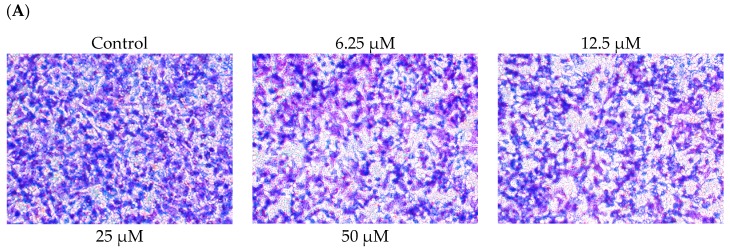

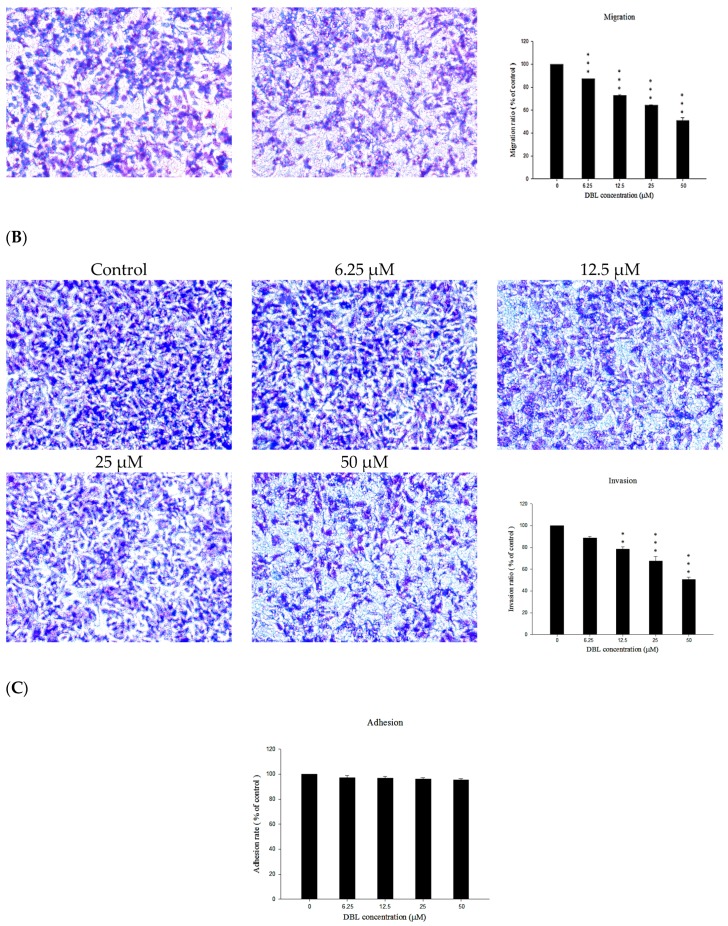
The effects of DBL on migration, invasion, and adhesion of A549 cells. The migration and invasion assays were assessed by passing A549 cells through 6.5 mm polycarbonate filters of 8 μm pore size. (**A**) Migration assay: A549 cells were treated with various concentrations (0, 6.25, 12.5, 25, and 50 μM) of DBL for 8 h; (**B**) Invasion assay: the upper chambers were coated with Matrigel. A549 cells were treated with DBL for 24 h. All of the chambers were fixed, stained, and photographed in 200× microscopic power field; (**C**) Adhesion assay: the A549 cells were treated with DBL for 24 h, then seed cells in Matrigel-coated 96-well plates. The adhesion rate was evaluated by MTT assay. Data represent mean ± SEM from three independent experiments. Statistical significance was analyzed by one-way ANOVA and post hoc test was Scheffe test. (** *p* < 0.01 and *** *p* < 0.001).

The ability to migrate through vessel endothelium and invade other tissue are characteristics of metastatic cancer cells. Therefore, to investigate whether DBL has an inhibitory effect on cancer metastasis, we conducted migration, invasion, and adhesion assay. First, to elucidate the migratory ability of A549 cells, we used A549 cells treated with the indicated concentrations of DBL prior to transwell assay. As shown in Figure 2A, migration was markedly inhibited in A549 cells. The inhibition rate of 50 μM DBL was 49.1% (*p* < 0.001) compared with control A549 cells. Next, we investigated the effects of DBL on A549 cells’ ability to invade freely through Matrigel to determine whether this ability could be inhibited by DBL. The results revealed that DBL suppressed cancer cell invasion ability in A549 cells (Figure 2B). The inhibition rate of 50 μM DBL was 49.3% (*p* < 0.001) compared with control group. Cancer cells make new contacts with the ECM after invading the host tissues. Hence, DBL was evaluated for inhibiting this effect via adhesion assay. Our result showed that DBL had no obvious effect on cell–matrix adhesion of A549 cells (Figure 2C). The above results suggested that DBL’s anti-metastasis activity may be by inhibiting the motility of cancer cells.

### 2.3. DBL Exerts Inhibitory Effects on Activity, Protein Expression, and mRNA Levels of MMPs

**Figure 3:**
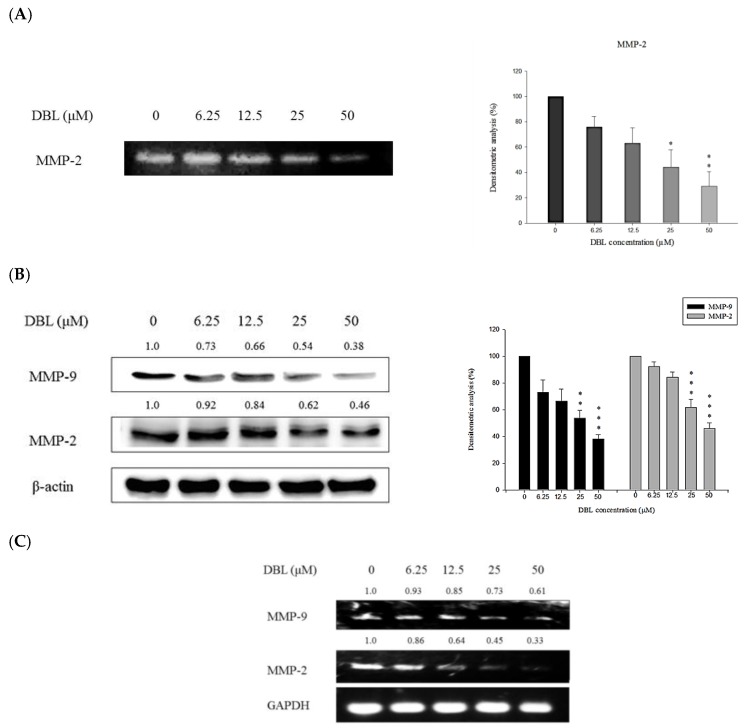
DBL attenuates activities, protein expression, and mRNA level of MMPs in A549 cells. A549 cells were treated with various concentrations of DBL. (**A**) Collected supernatants and gelatin zymography were used to analyze the activities of MMP-2; (**B**) protein expression of MMP-9 and MMP-2 was determined by Western blot; (**C**) mRNA levels of MMP-9 and MMP-2 were examined by RT-PCR. The data are presented as the mean ± SEM for three different experiments performed in triplicate. Statistical significance was analyzed by one-way ANOVA and post hoc test was Scheffe test. (* *p* < 0.05, ** *p* < 0.01, and *** *p* < 0.001 compared with control group.)

MMPs are crucial for degrading basement membrane. Among them, MMP-2 and -9 are paid a lot of attention in most researches. The activity, protein expression, and mRNA levels in A549 cells were evaluated by treating the cells with different concentrations of DBL and subsequent analysis using gelatin zymography, Western blot, and RT-PCR. Gelatin zymography analysis was used to examine the activity of MMP-2. As the results showed, enzymatic activity of MMP-2 was suppressed by DBL (Figure 3A). According to the Western blot results, the protein expression of MMP-2 and -9 is inhibited by DBL, and quantification analysis showed that the inhibition rates of 50 μM DBL are 62.0% and 54.0% for MMP-2 and -9, respectively, compared with control group (Figure 3B). Further, we also found DBL could affect mRNA levels of MMP-2 and -9. As shown in Figure 3C, the results of RT-PCR revealed DBL significantly reduced the MMP-2 mRNA level. Above results indicated the inhibitory effects of DBL on cancer cell metastasis may be due to the suppression of MMP-2 and -9 expression.

### 2.4. DBL Takes Part in Cancer Metastasis Suppression in Multiple Pathways, Revealed by Protein Expression

To investigate the possible mechanisms by which DBL inhibited cancer cell metastasis in vitro, we used Western blot to reveal proteins’ expression.

**Figure 4:**
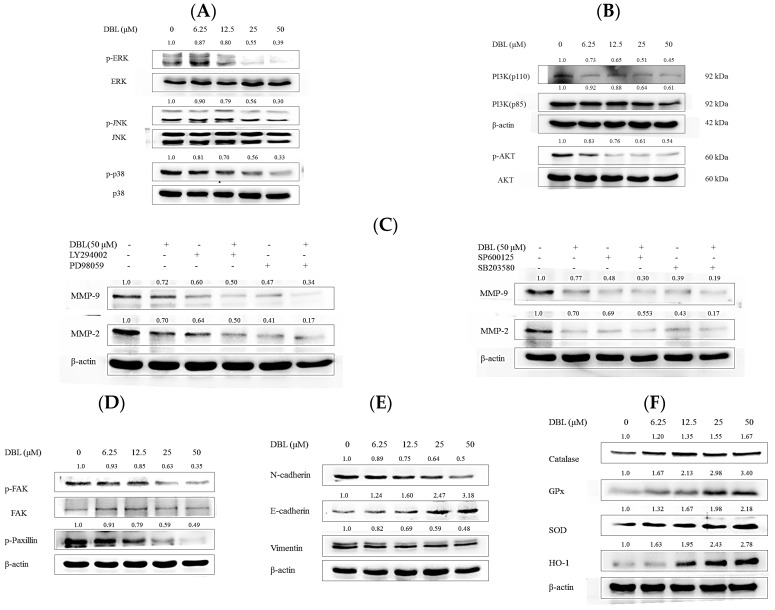
DBL-affected protein expression via Western blot in A549 cells. (**A**) The phosphorylation of mitogen-activated protein kinase (MAPK) (extracellular signal-regulated kinase (ERK), c-Jun N-terminal kinase (JNK), and p38); (**B**) phosphoinositide 3-kinase (PI3K)/AKT; and (**C**) matrix metalloproteinase (MMP)-2 and MMP-9 protein expression treated with PI3K inhibitor (LY294002), ERK inhibitor (PD98059), JNK inhibitor (SP600125), and p38 inhibitor (SB203580), or co-treated with DBL; (**D**) Phosphorylation of focal adhesion kinase (FAK) and paxillin; (**E**) epithelial to mesenchymal transition (EMT)-related protein including mesenchymal biomarkers (N-cadherin, vimentin) and epithelial biomarker (E-cadherin). (**F**) Antioxidant enzymes: catalase, glutathione peroxidase (GPx), superoxide dismutase (SOD), and heme oxygenase 1 (HO-1). A549 cells were treated with different concentrations of DBL for 24 h. Cell pellets were lysed with RIPA buffer. Quantitated proteins were separated by SDS-PAGE and conjugated with specific antibodies. The data are presented for three different experiments performed in triplicate.

First, PI3K/AKT/MAPKs/MMPs are responsible for degradation of ECM and basement membrane. As showed in Figure 4A,B, DBL inhibited MMP-2 and -9 expression while reducing the expression of phosphorylated forms of extracellular signal-regulated kinase (ERK), c-Jun N-terminal kinase (JNK), p38, AKT, and PI3K. However, the above total protein levels remained unchanged. To examine this hypothesis for MMPs, PI3K, and MAPKs, we added MEK inhibitor PD98059, PI3K inhibitor LY294002, JNK inhibitor SP600125, and p38 inhibitor SB203580 to A549 cells, with or without DBL treatment. In Figure 4C, the results show that when treated with each inhibitor and DBL, protein expression of MMP-2 and -9 have synergistic effects. Although there are some researches revealed that PI3K is associated with cell death or apoptosis. The supplementary result revealed that DBL inhibited protein expression of PARP. This result demonstrated DBL is not enhanced A549 cells apoptosis but endowed A549 cells metastasis. (Figure S1).

Next, focal adhesion kinase (FAK)/paxillin/Src is related with cancer cells’ migratory ability [21]. The results revealed that DBL inhibited the phosphorylated form of FAK and paxillin, while total protein expression of FAK and Src remained unchanged in A549 cells. Therefore, we speculated that DBL inhibition of cancer metastasis may be related with inhibition of migratory ability.

**Figure 5:**
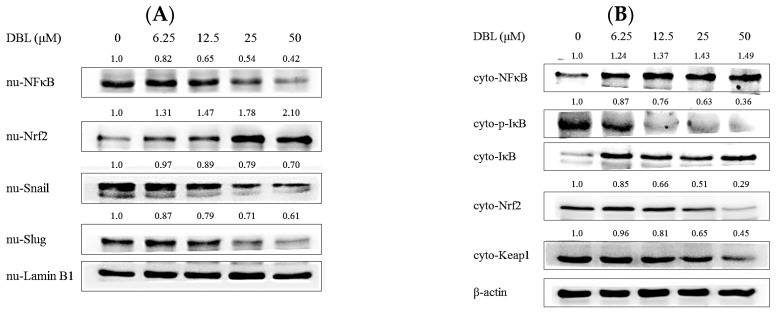
DBL affected translocated protein expression in A549 cells. (**A**) The protein expression of nuclear factor κB (NFκB), nuclear factor erythroid 2-related factor 2 (Nrf2), Snail, and Slug in nucleus. The internal control is laminB.1 (**B**) The protein expression of NFκB, phosphorylated inhibitor of NFκB (p-IκB), IκB, Nrf2, and kelch-like ECH-associated protein 1 (Keap1) in cytosolic fractions. A549 cells were treated with different concentrations of DBL for the appropriate time. Separated nuclear and cytosolic fraction were used with commercial product (Pierce Biotechnology, Rockford IL, USA, 200 reactions). Quantitated proteins were separated by SDS-PAGE and conjugated with specific antibodies. The data are presented as the mean ± SEM for three different experiments performed in triplicate.

Third, we examined whether DBL could affect major regulators and markers of epithelial to mesenchymal transition (EMT), which is an important step when cancer progression and metastasis [22]. Western blotting analysis results showed that DBL notably increased an epithelial marker (E-cadherin) and decreased the mesenchymal markers (N-cadherin and vimentin) (Figure 4E). Also, we found DBL repressed EMT-related transcription factors, Snail and Slug. The protein expression of Snail and Slug in nuclei was significantly inhibited by DBL (Figure 5A). Therefore, we can propose that DBL inhibited cancer cells metastasis by affecting EMT-related proteins.

Fourth, Nrf2/HO-1/antioxidant are enzymes related to ROS, which are important for metastasis because ROS could contribute to EMT and angiogenesis. [23] As the results revealed, DBL could activate antioxidant enzymes (SOD, GPx, catalase, and HO-1) in A549 cell (Figure 4F). Nrf2 played an important role, in which downstream antioxidant enzymes were mediated [24]. In the A549 cells, protein expression of Nrf2 in the nuclear fraction was elevated; in the cytosolic fraction, it was the contrary (Figure 5A,B).

Finally, we investigated protein expression of NFκB and inhibitor of NFκB (IκBα). NFκB is a transcription factor involved in many pathological processes, such as metastasis [15]. As shown in Figure 5A, the expression of NFκB in nuclear extracts was decreased by DBL treatment in A549 cells. DBL inhibited activated NFκB translocation from cytosol to nucleus. In the meantime, phosphorylated IκBα expression increased and non-phosphorylated IκBα expression decreased. Therefore, DBL may inhibit transcription factor NFκB and thus regulate downstream proteins’ expression—such as MAPKs, MMPs, and so on—to cause inhibition of cancer cell metastasis.

## 3. Discussion

Cancer metastasis includes numerous steps, such as epithelial to mesenchymal transition (EMT), degradation of extracellular matrix (ECM), and invasion of cancer cells [25]. In addition, tumor microenvironments, especially oxidative stress, is important in tumor progression and cancer metastasis [2]. The previous in vitro experiments demonstrated DBL had no obviously cytotoxicity and significantly inhibited the migratory and invasive abilities in A549 cells (Figure 2A,B). Therefore, we investigated the possible mechanisms by which DBL inhibits cancer cell metastasis.

First, many researches showed that high expression of MMP-2 and MMP-9 proteins are related with prognosis caused by cancer metastasis [26]. MMPs are involved in interactions between cancer cells and the ECM [27]. When the ECM is degraded, cancer cells detach from the primary tumor and cross barriers into arteries to cause cancer metastasis [12]. Therefore, inhibition of MMPs has been considered a crucial step in preventing cancer metastasis. Our results revealed DBL inhibited activities and protein expression, and decreased mRNA levels of MMP-2 and -9 (Figure 3).

There are endogenous specific inhibitors (e.g., tissue inhibitor of metalloproteinase (TIMP)) which bind to active MMPs [28]. TIMPs and MMPs form high-affinity 1:1 complexes to inhibit the activities of MMPs. Each TIMP varies in their tissue-specific expression and ability to inhibit different MMPs [13]. MMP-9 and -2 have a high affinity to TIMP-1 and -2, respectively [29]. It has been demonstrated that an imbalance between MMPs and TIMPs has been observed in breast carcinoma [30] and lung cancer. However, our results revealed that DBL did not obviously activate the protein expression of TIMP-1 and -2 (data not shown). Based on protein expression of MMP-2/-9 being regulated via complex molecular signaling pathways, next we investigated other mechanisms that also affect MMP-2/-9 or expression of other proteins related with the steps of cancer metastasis.

Research showed that many effects, such as proliferation and metastasis, are related with MAPK activation [31]. Additionally, in many types of cancer, MAPKs and PI3K/AKT have been illustrated to affect MMP secretion. Our results indicated that DBL and MAPK (PI3K, ERK, JNK, p38) inhibitor-treated A549 cells resulted in the protein expression of MMP-2 and -9 being more significantly inhibited than when only treated with a single inhibitor and DBL. PI3K activation has been demonstrated, which can affect downstream target AKT and stimulate the protein’s expression [32]. In different cancer cell types, PI3K/AKT has been proved to be related with cancer metastasis through regulating activities of MMP-2 and -9 [33,34]. Moreover, MAPKs, especially ERK, also proved to inhibit specific cytoskeletal and focal adhesion proteins, which correlated with cancer cells’ migratory ability [31].

Recent reports revealed repression of FAK/paxillin/Rac/MMP downstream signaling pathways could effectively inhibit the invasive ability of cancer cells [21]. FAK is a non-receptor tyrosine kinase which affects cells’ migratory ability and Snail-1-dependent EMT [35]. In various metastatic cancer cell types, the essential condition for cell migration is paxillin, a focal adhesion molecule which binds with FAK [36]. The results showed that DBL markedly inhibited the phosphorylation state of FAK and paxillin (Figure 4D). Therefore, we propose DBL inhibits lung cancer cell line A549 metastasis through inhibition of the FAK/paxillin signaling pathway. On the other hand, epithelial to mesenchymal transition (EMT) is a crucial step in cancer metastasis because EMT is a phenotype which causes cancer cell migration and invasion by breaking cell–cell junctions [25]. The switch between epithelial biomarker and mesenchymal biomarker happens in the EMT process [37]. This switch includes downregulating E-cadherin and increasing vimentin and N-cadherin [35,38]. Snail and Slug are transcriptional factors which correlate with the EMT process. These transcriptional factors have been demonstrated to affect E-cadherin expression and allow cancer cells to maintain an invasive phenotype [39]. Our results revealed that DBL repressed Snail and Slug protein expression in the nuclear fraction, and mesenchymal biomarkers vimentin and N-cadherin. Furthermore, DBL increased the protein expression of E-cadherin, which is a well-known epithelial biomarker. These results suggested DBL inhibited A549 cells invasion and migration by suppressing the EMT process.

In the past decade, reactive oxygen species (ROS) have been investigated for their role in tumor progression, and research has also indicated that ROS markedly stimulates the EMT process and migratory ability in many cancer cell types [7]. Normal intracellular metabolism and environmental stimuli can cause ROS generation. Detoxification enzymes—include HO-1, SOD, catalase, and GPx—can neutralize these oxidative stresses [40]. SOD is an enzyme that can convert the superoxide radical to hydrogen peroxide. Subsequently, catalase and GPx transform hydrogen peroxide to water, then lead to repression of excess ROS [41]. In this way, our results revealed DBL significantly activated protein expression of these antioxidant enzymes and ameliorated cancer metastasis caused by the imbalance of ROS (Figure 4F). Besides AP-1 and NFκB, the kelch-like ECH-associated protein 1 (Keap 1) and nuclear factor erythroid 2-related factor 2 (Nrf2) pathways have been considered to play pivotal roles in regulating detoxification enzymes and antioxidant genes [42]. After oxidative stress stimuli, Nrf2 dissociates and translocates to the nucleus, then promotes detoxification enzymes to achieve cytoprotective effects [24]. After treatment with DBL, the protein expression of Nrf2 in the nuclear fraction was activated, which agreed with increased protein expressions of antioxidant enzymes, previously (Figure 5).

In addition to Nrf2, Snail, and Slug, another transcriptional factor, NFκB, is worth of noting. Without stimuli, NFκB proteins are constitutes of p65, p50, and IκBα, thus existing as an inactive form in the cytoplasm [43]. In cancer microenvironments, the inhibitor of NFκB (IκBα) is phosphorylated and ubiquitinated, leading to a p65–p50 heterodimer translocating into the nucleus and binding with specific consensus sequences to activate downstream signaling transduction, including MMPs [44]. Hence, NFκB has been considered as an important transcriptional factor in tumor progression and metastasis. Although there is a research pointing to DBL not being a direct inhibitor of IκBα kinase (IKK), IKK forms a complex with IκBα and leads to the inhibition of IκBα phosphorylation [19]. DBL inhibition of NFκB protein expression in the nucleus may be due to interference of upstream regulatory kinases such as PI3K/AKT, MAPK, and so on. In addition, ROS is another promoter causing NFκB activation [7]. DBL significantly elevated antioxidant enzymes to decrease content of ROS, which also may cause NFκB downregulation.

## 4. Materials and Methods

### 4.1. Chemical Reagents and Antibodies

F-12 Nutrient Mixture (Ham), trypsin-EDTA, fetal bovine serum (FBS), penicillin/streptomycin (PS), and other culture supplies were obtained from Gibco (BRL life Technologies, Grand Island, NY, USA). 3-(4,5-Dimethylthiazolyl-2)-2,5 diphenyltetrazolium bromide (MTT), and other chemical reagents such as Tris, sodium dodecyl sulfate (SDS), and gelatin were of analytical grade and purchased from Sigma-Aldrich Chemical Co. (St. Louis, MO, USA). Matrigel was purchased from BD Sciences (San Jose, CA, USA). Antibodies against phosphorylated and non-phosphorylated forms of IκBα, NFκB, JNK, and ERK were purchased from Cell Signaling Technology (Bevely, MA, USA). Antibodies of Lamin B1, MMP-2, MMP-9, AKT, catalase, GPx, and SOD were from GeneTex Inc. (San Antonio, TX, USA). Other antibodies, polyvinylidene fluoride transfer membranes (Immobilone P), and ECL were purchased from Millipore Corp. (Bedford, MA, USA).

### 4.2. Cell Lines and Cell Culture

The human A549 lung adenocarcinoma cell lines were obtained from Food Industry Research and Development Institute, Hsin Chu, Taiwan. A549 cells were cultured in F-12 medium and cultured medium containing 10% (*v*:*v*) FBS and penicillin/streptomycin (100 U/mL) in a humidified incubator under 5% CO_2_ at 37 °C.

### 4.3. Isolation and Determination of DBL

DBL was isolated from fruiting body of *P. linteus* (PL), which was dried, powdered, and extracted with 95% ethanol, and partitioned with the ethyl acetate (EA) fraction. The details of isolation are as follows. PL powder was extracted with 95% ethanol at room temperature three times. Each extract was combined and filtered, and then evaporated at 40 °C (N-11, Eyela, Japan) to dryness under reduced pressure to obtain a dark-brown residue. The crude extract was suspended in H_2_O, and then partitioned with *n*-hexane (×2), EA (×2), and *n*-butanol (×2), successively. It yielded five fractions, and DBL was purified from the EA soluble portion. This portion of the EA fraction was subjected to silica gel chromatography using stepwise CHCl_3_–MeOH (9:1, 8:2, 1:1 *v*/*v*) as eluent. Final purification was achieved by preparative HPLC (Spherisorb ODS-2 RP18, 5 μm (Promochem) 250 × 25 mm, acetonitrile–H_2_O (83:17 *v*/*v*), at a flow rate of 10 μL/min and UV detection at 375 nm). Fraction was recrystallized from EA to obtain DBL.

### 4.4. Cell Viability Assay

A549 cells were seeded at a density of 2 × 10^4^ cells per well in 96-well plates and incubated for 24 h. Then, cells were treated with various concentrations of DBL (0, 6.25, 12.5, 25, 50 μM) for 24 and 48 h. After the exposure periods, the medium was replaced with MTT and incubated at 37 °C for 4 h. Isopropanol/HCl solution was used to dissolve the formazan crystals. The absorbance was measured spectrophotometrically at 570 nm. The cell viability was calculated and compared with control group.

### 4.5. Gelatin Zymography Assay

MMP-2 activities in cells culture supernatants were evaluated by gelatin zymography according to the protocol developed by Kleiner and Stetler-Stevenson with minor modification [45]. Briefly, A549 cells (1 × 10^6^) were seeded into 6-well plates and treated with the indicated concentrations of DBL for 24 h. The culture supernatants were harvested and electrophoresed in an 8% SDS-PAGE gel containing 0.1% gelatin. After electrophoresis, gels were washed with 2.5% Triton X-100 buffer and incubated with reaction buffer for enzymatic reaction containing 1% NaN_3_, 10 mM CaCl_3_, and 40 mM Tris-HCl (pH 8) for at least overnight at 37 °C. The gels were stained with Coomassie blue and destained in 10% acetic acid (*v*/*v*) and 40% methanol (*v*/*v*). The relative MMP-2 activities were quantified by Kodak Molecular Imaging software (Version 4.0.5, Eastman Kodak, Rochester, NY, USA).

### 4.6. Cell Migration and Invasion Assay

The migratory ability of A549 cells to pass through 6.5 mm polycarbonate filters of 8 μm pore size was modified from Repesh [46]. A549 cells were suspended in serum-free medium containing indicated concentrations of DBL in the upper transwell chambers. The lower compartments were loaded with 10% FBS medium as a chemoattractant. The difference between cell migration and invasion is that the filters were coated with Matrigel to form thin continuous films in the invasion assay. After the appropriate time period, cells in the upper surface of the filter were removed with cotton swabs, and cells that had invaded across to the lower surface of the filter were fixed and stained with Giemsa solution. The stained cells were observed under a light microscope and quantified by manual counting. For each replicate, six randomly selected fields were analyzed for each group.

### 4.7. Cell Adhesion Assay

A549 cells were treated with various concentrations of DBL for 24 h. After that, cells were collected, cell number adjusted to 1 × 10^4^ cells/mL, and placed on 96-well plates coated with Matrigel overnight. After incubation for the appropriate time, nonadherent cells were washed twice by phosphate-buffered saline (PBS). The attached cells were evaluated by MTT assay.

### 4.8. Reverse Transcription-Polymerase Chain Reaction (RT-PCR)

According to the Gene JET RNA Purification kit’s instructions, total RNA from DBL-treated A549 cells was purified and extracted for the next experiment. Reverse transcription for the synthesis of 1 μg total RNA to cDNA was performed using the cDNA Synthesis kit as per the manufacturer’s protocols. The cDNA was amplified by PCR with the following primer: MMP-9, 5′-CGGAGCACGGAGACGGGTAT-3′ (sense) and 5′-TGAAGGGGAAGACGCACAGC-3′ (antisense); MMP-2, 5′-GGCCCTGTCACTCCTGAGAT-3′ (sense) and 5′-GGCATCCAGGTTATCGGGGA-3′ (antisense); GAPDH, 5′-CGGAGTCAACGGATTTGGTCGTAT-3′ (sense) and 5′-AGCCTTCTCCATGGTGGTGAAGAC-3′ (antisense). PCR products were analyzed by agarose gel electrophoresis and visualized by stained with ethidium bromide (EtBr).

### 4.9. Nuclear and Cytosolic Protein Extraction

A549 cells were treated indicated concentrations of DBL. After appropriate times, cells were harvested and nuclear/cytosolic protein extracts prepared according to the manufacturer’s protocol (Pierce Biotechnology, 200 reactions).

### 4.10. Western Blot Analysis

The protein expressions of A549 cells were measured by Western blotting. The cells were harvested after pretreatment of decided concentrations of DBL for 24 h and lysed with ice-cold RIPA buffer (containing 1% NP-40, 50 mM Tris base, 0.1% SDS, 0.5% deoxycholic acid, 150 mM NaCl, pH 7.5). Protein concentration was measured using a standard bovine serum albumin (BSA) curve. Sample containing equal amounts of protein (50 μg), as indicated, were separated by SDS polyacrylamide gel electrophoresis and transferred to PVDF membranes. Nonspecific binding of the membranes was blocked with blocking buffer (10% non-fat skim milk/1 × TBS/0.5% Tween-20) for 1 h at room temperature. The membranes were hybridized with appropriate dilution of specific primary antibodies overnight at 4 °C and then washed three times before incubating with the horseradish peroxidase (HRP)-conjugated secondary antibodies at appropriate dilution for 1 h at room temperature. The bands were visualized by a chemiluminescence (ECL) detection kit.

### 4.11. Statistical Analysis

Values were obtained from at least three independent experiments and difference between each group were analyzed using one-way analysis of variance (ANOVA) and Scheffe’s post hoc test. The significance of differences is considered a *p* value < 0.05.

## 5. Conclusions

**Figure 6:**
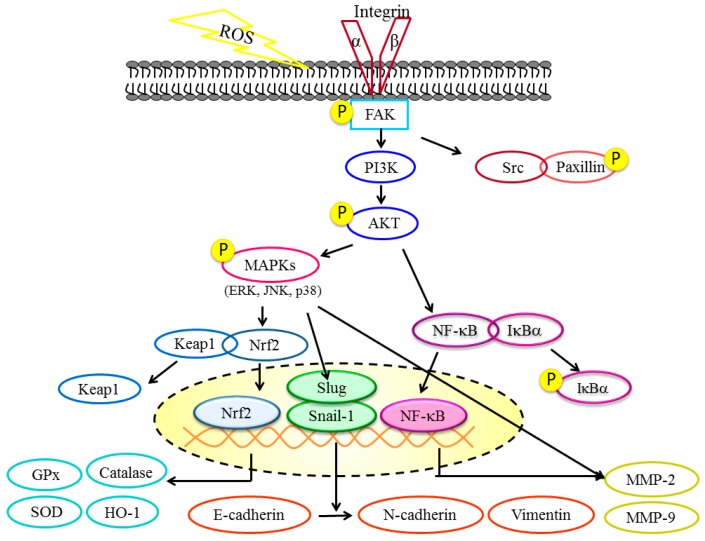
The mechanisms of DBL inhibit human non-small cell lung carcinoma cells metastasis.

Overall, the above results revealed that DBL inhibits migratory and invasive abilities in A549 cells. The anti-metastasis efficacy may due to DBL regulating several pathways. First, DBL markedly inhibited MMP-2 and -9 enzymatic activities and protein expression. MMPs play a crucial role in cancer metastasis. Then, our results indicated PI3K/AKT/MAPK pathways regulated MMP-2 and -9, and these pathways are also regulated by DBL. Further, DBL inhibited the FAK/paxillin signaling pathway, thereby regulating the migratory ability of A549 cells. Other signaling pathways, such as Nrf2/HO-1/antioxidant enzymes and EMT-related proteins, were also regulated by DBL. Most importantly, DBL inhibited transcriptional factor NFκB, which mediates many cancer metastatic characteristics, such as MMP-2 and -9 activities (Figure 6). Collectively, our results demonstrated the potential of DBL to suppress lung cancer cell metastasis. Although more evidence and animal models are needed to investigate the anti-metastatic effects for human therapy, our study provides preliminary results for a promising strategy in preventing lung cancer metastasis.

## Supplementary Materials

Supplementary materials are available online. Figure S1: DBL inhibited PARP protein expressions via Western blot in A549 cells.
