# Supplementary material for: 3,4-Dihydroxybenzalactone Suppresses Human Non-Small Cell Lung Carcinoma Cells Metastasis via Suppression of Epithelial to Mesenchymal Transition, ROS-Mediated PI3K/AKT/MAPK/MMP and NFκB Signaling Pathways
Source: Molecules. 2017 Mar 28;22(4):537. doi: 10.3390/molecules22040537 (PMC6154291; doi:10.3390/molecules22040537)
Supplement: Supplementary file 1 [file molecules-22-00537-s001.pdf]

Supplementary information

Fig S1

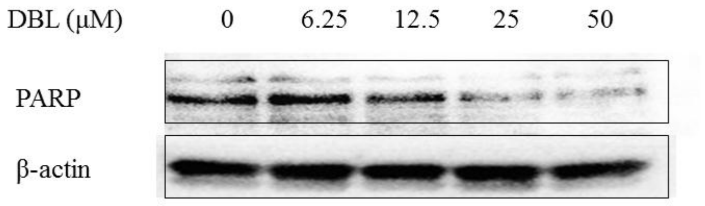

Figure 1S. DBL inhibited PARP protein expressions via western blot in A549 cells. A549 cells were treated with different concentrations of DBL for 24h. Cell pellets were lysis with RIPA buffer. Quantitive proteins were separated by SDS–PAGE and conjugated with specific antibodies.
